# Ionophore application for artificial oocyte activation and its potential effect on morphokinetics: a sibling oocyte study

**DOI:** 10.1007/s10815-021-02338-3

**Published:** 2021-10-13

**Authors:** Omar Shebl, Philip Sebastian Trautner, Sabine Enengl, Elisabeth Reiter, Christina Allerstorfer, Tamara Rechberger, Peter Oppelt, Thomas Ebner

**Affiliations:** Department of Gynecology, Obstetrics, and Gynecological Endocrinology, Kepler University Hospital, MedCampus IV, Krankenhausstr. 26-30, A-4020 Linz, Upper Austria Austria

**Keywords:** Artificial oocyte activation, Calcimycin, Ionophore, Morphokinetics, Time-lapse imaging

## Abstract

**Purpose:**

To evaluate whether ionophore application at the oocyte stage changes the morphokinetics of the associated embryos in cases of artificial oocyte activation.

**Methods:**

In a prospective sibling oocyte approach, 78 ICSI patients with suspected fertilization problems had half of their MII-oocytes treated with a ready-to-use ionophore (calcimycin) immediately following ICSI (study group). Untreated ICSI eggs served as the control group. Primary analyses focused on morphokinetic behavior and the presence of irregular cleavages. The rates of fertilization, utilization, pregnancy, and live birth rate were also evaluated.

**Results:**

Ionophore-treated oocytes showed a significantly earlier formation of pronuclei (t2PNa) and a better synchronized third cell cycle (s3) (*P* < .05). The rate of irregular cleavage was unaffected (*P* > .05). Ionophore treatment significantly improved the overall rates of fertilization (*P* < .01) and blastocyst utilization (*P* < .05).

**Conclusion:**

Ionophore application does not negatively affect cleavage timing nor is it associated with irregular cleavage.

## Introduction

One of the new technologies in the field of medically assisted reproduction from the last decade [1] is time-lapse technology (TLT). Since its development, TLT has shifted from an observational approach to a tool involved in the selection and prediction of embryo growth. To date, there are no randomized controlled trials supporting the clinical relevance of TLT [2], although this lack of evidence has not remained unchallenged [3, 4]. However, regardless of the potential causative impact on reproductive outcome, TLT has several advantages that would justify its use.

First, time-lapse incubators guarantee the safest and most stable embryo culture environment available by simply avoiding extensive exposure outside the incubator [5]. Second, continued embryo monitoring that allows for a 24-h surveillance of the oocytes, zygotes, and embryos facilitates the detection or further investigation of previously unknown phenomena in preimplantation development [6–8]. The good practice recommendation paper for the use of TLT by the ESHRE working group on time-lapse technology [9] further highlighted that TLT allows for a more flexible and better management of a laboratory workload.

Finally, TLT is an important tool within the framework of existing quality management systems. In fact, it may be used to train embryologists and assess intra- and inter-operator variability with respect to the annotation of morphokinetic parameters [10–12]. More importantly, TLT can also be used to identify and monitor in vitro stress that may arise from suboptimal culture conditions. In particular, a large number of consumables or new culture media for testing could interfere with anticipated cleavage timings [13, 14]. Although subtle changes may have no clinical consequences [14, 15], larger deviations in morphokinetic behavior may reflect severe physiological problems [16].

In other words, in the course of the validation of a new process in an IVF laboratory [17], TLT could be an effective tool to support the capability and safety of the same. One such process [18–20] is assisted or artificial oocyte activation (AOA). To date, AOA has been successfully used to artificially induce intracellular calcium (potentially stimulating activation and mitosis) in cases of fertilization failure, fertilization problems (< 30%), and severe male factor infertility, and in cases with impaired embryo development.

In this context, information on the potential effects of AOA, for example application of an ionophore on cleavage behavior, is scarce and primarily based on preliminary data from posters [21, 22] or oral presentations [23]. Recently, Martínez et al. [24] were the first to evaluate the association between AOA and morphokinetics in donor oocyte cycles of patients with a known mutation in the PLCζ1 gene in great detail.

This study aimed to identify any potential influence of a ready-to-use ionophore (calcimycin) on cleavage timings and synchronicity. In order to find an optimally matched control group, a sibling oocyte approach was chosen, with half of the eggs being treated with AOA and the other half not treated.

## Material and methods

### Patients

In the 3-year study period (2017–2019), a total of 78 patients were included in this prospective observational study with retrospective analysis of morphokinetic data (ethical votum #1115/2021). All patients provided informed consent and accepted that for approximately half of their mature gametes, oocyte activation was artificially supported by calcimycin, which is a known Ca^2+^-ionophore. This additional support was offered due to a history of borderline fertilization (*n* = 47) defined as < 50% [25] or suspected severe male factor infertility (*n* = 31) [26]. The patients were not charged for this AOA technique because of the study conditions. Every patient was only included once since the purpose of the split inophore cycle was diagnostic. Based on the results of the present sibling oocyte approach, a subsequent cycle would either be without ionophore application or with all eggs receiving AOA.

The female mean age was 32.9 ± 4.1 years, and the patients suffered from endometriosis (9.0%), PCOS (6.4%), or bilateral tubal factor (1.3%). Apart from four cases of unexplained infertility (5.1%), all couples had an additional male factor (94.9%). Most of the stimulations were performed according to an antagonist protocol (*n* = 52, 69.2%), whereas the remaining cycles were treated with a long protocol. Ovulation induction was always performed with hCG when the lead follicle(s) reached a diameter of 18–20 mm and serum estradiol level was adequate (approximately 150 pg/ml E2 follicle seen on ultrasound). Subsequently, ovarian puncture was performed transvaginally using an ultrasound 36 h after ovulation induction.

### ICSI, AOA, and embryo culture

Cumulus-oocyte complexes (COCs) were collected from GM501 Wash (Gynemed, Lensahn, Germany). After a 2–3 h resting period in a big box incubator (Labo C201, Labotect, Göttingen, Germany), COCs were carefully denuded (combining enzymatic and mechanical steps). Oocytes that were identified as mature (MII) were injected using Microtech pipettes (Gynemed) and a Luigs and Neumann micromanipulator (Ratingen, Germany). Untreated control oocytes were immediately placed into a special time-lapse culture dish (CultureCoin®, Esco Medical Technologies) for use in the time-lapse system Miri TL (Esco Medical Technologies, 6.5% CO_2_, 5% O_2_). The other half of the MII-oocytes were treated with a ready-to-use ionophore compound (GM508 CultActive, Gynemed) within 10 min after ICSI, as published earlier [26, 27]. Since GM508 CultActive is a bicarbonate-buffered product, it requires preincubation in a CO_2_ atmosphere (4 h according to the supplier). This process is usually performed in a 4-well dish in which one well contains a droplet of GM508 CultActive (30–50 µl in our hands) and three wells contain washing medium (GM501 Wash). All agents must be covered with mineral oil. It is recommended to agitate the bottle with GM508 CultActive before use in order to avoid precipitation.

After 15 min of incubation in GM508 CultActive and a thorough washing process, the oocytes subjected to AOA were transferred to the same CultureCoin. Notably, the ionophore used in the ready-to-use product was calcimycin, also known as A23187.

Although video sequences were automatically generated, static morphological assessments were performed at fixed time points [28]. It should be noted that on day 1 (circa 22 h post ICSI), the videos were screened for the extrusion of the second polar body and the presence of two pronuclei so that fertilization was never missed. This led to the scenario in which the number of fertilizations equals the number of cleaved embryos. Good-quality embryos on days 2–3 showed a stage-appropriate cell number, with less than 10% of fragments showing no signs of multinucleation. It should be clarified that it was not screened for multinucleated cells and, thus, the fact that there were multinucleated cells at time points [29] other than those suggested by Alpha and ESHRE cannot be overlooked [28]. Any “evidence of compaction that involves virtually the entire embryo volume” on day 4 was considered to be a proper development and reflected good quality [28]. Blastocyst quality was scored on day 4 [30] or day 5 according to usual criteria such as grade of expansion and quality of the two cell lineages, namely trophectoderm (TE) and inner cell mass (ICM).

Whenever possible, blastocysts were transferred, and this was performed in the majority of cases (*n* = 60, 76.9%). When blastocysts were formed on day 4 and transfer (and/or vitrification) was performed on the same day, all surplus embryos (non-blastocysts on day 4) were cultured for an additional day in order to allow for day 5 blastocyst evaluation [30].

Apart from four women who had a mixed double embryo transfer, all patients had either embryos transferred exclusively from the untreated group or from the ionophore group. All but one of these (*n* = 73) had a single embryo transfer, and in cases where blastocyst transfer was performed, it was an elective single (blastocyst) transfer. All transfers were performed under ultrasound guidance with the help of a Semtrac soft catheter set 4.2 (Gynetics, Lommel, Belgium).

### Time-lapse annotation

Notably, the starting time of the videos was set at the mid-time of microinjection [13] so that all videos (AOA and non-AOA) were synchronized. The shortest possible image frequency was chosen (5 min, seven focal planes) to avoid missing any cellular events of interest. Morphokinetic parameters were annotated by a single embryologist (T. E.) according to previously published criteria [13]. In detail, early morphokinetic parameters such as t2PB, t2PNa, and 2PNf, as well as later cleavage events (t2-t8, tSC, tSB, tB), were taken into consideration. According to the definition in [13], these events are marked either by the first frame in which a certain morphological feature cannot be visualized any longer (t2PNf) or the first frame that shows either a distinct separation of cells (e.g., t2PB, t2-t8) or by the initiation of a certain process (tSB).

In addition, dynamic cell cycle variables (duration and synchronicity of embryonic cell cycles) according to Ciray et al. [13] were automatically calculated using the Miri TL software (s2, s3). Special care was taken to identify the embryos with irregular cleavages. In this study, the nomenclature from Ciray et al. [13] was used. In detail, the samples were screened for rapid cleavage (t3–t2 < 5 h), trichotomous mitoses (e.g., t3–t2 = 0), cell fusions (reduction of blastomere number at any stage), and the presence of planar embryos [31]. Slight modifications were applied for trichotomous cleavage events, as the trichotomous mitoses were further subdivided according to the embryonic cell cycle. In other words, we not only focused on the zygote stage but also on the 2- and 4-cell embryo stages. Finally, irregular chaotic division (ICD) was characterized by the inability to divide clearly into separate blastomeres. Desai et al. [32] found that in “such embryos, the process of cleaving can be prolonged, eventually resulting in an embryo with irregular blastomeres and large cellular fragments.”

### Statistics

Seventeen days after intrauterine transfer, the blood concentration of hCG was measured (biochemical pregnancy). Clinical pregnancy was determined by visualization of at least one gestational sac with positive heart activity a month after embryo transfer. A subclinical pregnancy showed no fetal heartbeat. The implantation rate was defined by ultrasound visualization of a gestational sac per transferred embryo (subclinical pregnancies were included). Neonatal and perinatal outcomes were either obtained from an in-house registry or were requested from peripheral maternity clinics. Additional information was received from our neonatal intensive care unit (in the case of preterm delivery) and from the pediatric unit (in the case of minor malformations).

Using the Kolmogorov–Smirnov test, all variables were tested for a normal distribution. According to the outcome of the distribution, either a paired sample Wilcoxon sign rank test (no normal distribution) or a *t*-test for paired samples (normal distribution) was applied. Both tests involve paired data (control and study oocytes/embryos) to reduce the effect of confounders and to increase the test power. In other words, every individual parameter (e.g., rates of fertilization and blastocyst formation, t2PN, tPNa, tPNf) was analyzed on a per-patient basis.

The primary endpoint of the study was the comparison of the exact timing of morphokinetic events in study and control oocytes leading up to full blastocyst formation (tB). The secondary endpoints included the presence of irregular cleavages and the rates of fertilization and blastulation. In addition, the focus was on utilization rate, clinical pregnancy rate, and live birth rate.

It is very likely that developmental arrest of cleavage stage embryos may be adumbrated by an insidious cleavage delay at earlier phases. In order to account for this confounding factor, statistical analysis was not only done for the entirety of embryos but also for the subgroup of embryos that entered the blastocyst stage (and in which imminent growth delay or arrest was not a major issue). Bootstrapping method (2000 repeates) was used to calculate 95% CIs. However, in all cases, statistical significance was set at *P* < 0.05.

## Results

A total of 939 COCs were collected from 78 patients undergoing ICSI. After the denudation process, 747 oocytes (79.6%) were found to be mature (mean ± SD: 9.6 ± 4.3 MII oocytes), i.e., at metaphase II. These MII-eggs were then injected, which led to 551 zygotes (73.8%). Minor percentages were found to be degenerated after ICSI (1.7%), parthenogenetically activated (1PN, 1.7%), or showed 3PN (2.3%). Table 1 indicates that the overall fertilization rate was significantly higher (*P* < 0.01, Wilcoxon test) in the oocytes that had been treated with ionophore (78.2%) than in the untreated controls (69.3%). Interestingly, ionophore application was not related to parthenogenetic activation or degeneration after ICSI in the present study.

**Table 1:** ICSI performance and blastocyst development in sibling oocytes of 78 patients either untreated or artificially activated with a ready-to-use ionophore compound

	Untreated oocytes	Ready-to-use ionophore
*N*	371	376
Degenerated	7 (1.9)	6 (1.6)
0PN^a^	93 (25.1)	60 (16.0)
1PN	8 (2.1)	5 (1.3)
2Pn^b^	257 (69.3)	294 (78.2)
3Pn	6 (1.6)	11 (2.9)
Good quality embryos d2	102 (39.7)	121 (41.1)
Good quality embryos d3	96 (37.4)	115 (39.1)
Embryos d5 culture	230	249
Day 4 compaction	111/230 (48.3)	130/249 (52.2)
Blastocyst formation	139 (60.4)	166 (66.7)
Utilization rate ^c^	111 (48.2)	136 (54.6)

^a,b^*P* < .01

^c^*P* < .05

Values in parentheses are percentages

Of all day 2 embryos, 40.5% were of good quality, and a similar rate was observed for day 3 (38.3%). The next day, 50.3% (241/479) showed signs of compaction. Finally, on day 5, blastulation was observed in 305 embryos (63.7%), 247 (81.0%) of which were of good quality so that they could be transferred or vitrified. Embryo/blastocyst quality was not affected by ionophore application (Table 1). On average, a 6% higher blastocyst formation rate in the ionophore-treated cohort did not reach the level of significance. (*P* = 0.063, Wilcoxon test).

**Fig. 1:**
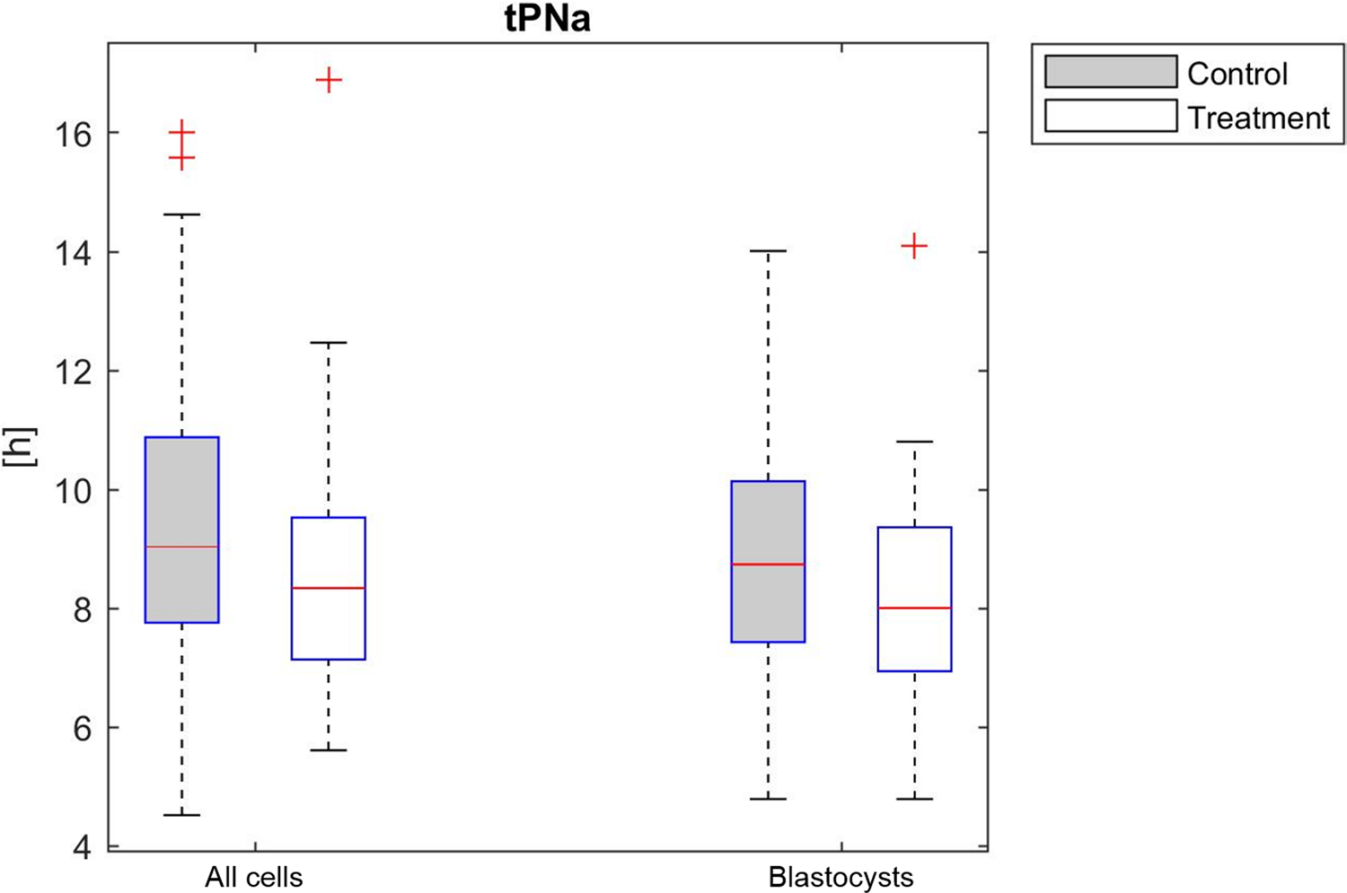
Box plot of timing of pronuclear appearance (*h* post t0) in study and control oocytes. Significant differences were observed (*P* < .05) in all oocytes (left side of the panel) and in only those which made it to blastocyst stage (right side). The box represents the interquartile range; the line inside the box represents the median. Outliers are shown using a plus symbol

The only cleavage timing that was significantly affected (*P* = 0.002) by the ionophore application was the appearance of the two pronuclei (t2PNa), while the remaining cleavage timings showed no correlation with AOA (Fig. 1, Table 2). The developmental benefit was still observable if only those oocytes were considered for calculation, which made it to the blastocyst stage (*P* = 0.02). Notably, the synchronicity of the four blastomere divisions within embryonic cell cycle 3 (referred to as s3) was significantly shortened in the ionophore group (*P* = 0.036). Again, analyzing only those embryos that made it to day 5 did not change the results (*P* = 0.048). Table 3 highlights that the application of an ionophore was not associated with the occurrence of irregular cleavages.

**Table 2:** Annotated morphokinetic parameters of all embryos that made it to the blastocyst stage in the study (ICSI-AOA) and control group (ICSI)

	Study group		Control group		Paired differences (95% CI)			*P*-value
Parameters	Mean	Range	Mean	Range	Mean	Lower bound	Upper bound	
t2PB	3.3	0.8–6.6	3.5	1.0–11.2	0.30	− 0.13	0.77	0.30
tPNa	8.2	4.8–14.1	8.9	4.8–14.0	0.74	0.28	1.25	0.02
tPNf	24.2	18.4–31.2	24.4	19.1–31.5	0.20	− 0.56	0.95	0.54
t2	27.1	21.0–34.2	27.6	20.7–38.2	0.50	− 0.41	1.44	0.32
t3	37.7	25.2–47.0	38.0	25.1–44.6	0.14	− 1.05	1.29	0.67
t4	39.3	31.7–50.2	39.9	31.9–51.3	0.50	− 0.52	1.53	0.31
t5	50.9	38.9–62.7	51.4	38.9–65.3	0.24	− 1.32	1.89	0.28
t6	53.0	43.5–66.3	54.0	41.8–72.5	0.92	− 0.62	2.44	0.29
t7	57.0	44.4–75.0	57.5	44.5–80.9	0.68	− 1.24	2.54	0.47
t8	63.9	48.0–84.7	65.5	47.5–90.1	0.67	− 0.45	1.28	0.12
tSC	83.7	65.1–102.8	83.7	58.1–103.1	1.7	− 0.42	3.89	0.53
tSB	99.3	71.9–115.6	99.3	75.4–116.6	0.5	− 1.96	2.91	0.58
tB	108.2	91.8–129.1	107.6	92.8–125.6	1.9	1.86	− 0.50	0.12

95% confidence intervals (CIs) were calculated by bootstrapping (2000 repeats), *P*-values by paired sample Wilcoxon sign rank test or *t*-test for paired samples

**Table 3:** Irregular cleavages in ionophore-treated and untreated sibling oocytes

	Untreated oocytes	Ionophor-treated
*N* fertilizations	257	294
Irregular chaotic division	29 (11.3)	22 (7.5)
Rapid cleavage	3 (1.2)	2 (0.7)
Trichotomous mitosis	17 (6.6)	18 (6.1)
1 to 3 cells	12 (4.7)	10 (3.4)
2 to 4 cells, 3 to 5 cells	5 (1.9)	8 (2.7)
Cell fusion	5 (2.0)	7 (2.4)
Planar embryos	2 (0.8)	2 (0.7)
Regularly cleaved embryos	201 (78.2)	243 (82.7)

Values in parentheses are percentages. *Pn* pronuclei. Irregular cleavages were assessed according to Ciray et al. (2014) and Desai et al. (2018). *P* > 0.05

Including those four patients with mixed embryo transfers, 83 embryos were transferred to 78 patients. The overall implantation rate was 50.6% (42/83). Positive hCG levels were measured in 42 out of 78 (53.8%) patients. Pregnanices were only achieved in patients who had either embryos from the study group (*n* = 45) or from the control group (*n* = 29) transferred (Table 4). However, one extrauterine pregnancy (2.4%) and seven pregnancy losses (16.7%) reduced the clinical pregnancy rate to 43.6%. One late pregnancy loss due to an infection in gestation week 16 (ionophore group) was observed. Consequently, the overall live birth rate was 42.3% (33/78). Since two twin (monochorionic-diamniotic) pregnancies (one in each group) were achieved (4.8%), 35 babies were born. There was one minor malformation per group, for example, a bilateral preauricular adnexa in the untreated group and a small femoral hemangioma in the ionophore counterpart. According to Table 4, there was no effect of the ionophore treatment on pregnancy outcome since the observed 10% higher live birth rate did not reach the level of significance. The same holds true for neonatal outcomes, which was the same for both groups.

**Table 4:** Pregnancy and neonatal outcome in 78 study patients divided into transfers from untreated and ionophore-treated embryos. Four patients had a mixed double embryo transfer (no pregnancies) and associated data are not included in this table

	Untreated	Ready-to-use ionophore
N homogeneous transfers	29	45
Embryos transferred	30	45
Positive hCG	14 (48.3)	28 (62.2)
Clinical pregnancy	11 (37.9)	23 (51.1)
Extrauterine pregnancy	0	1 (2.2)
Live birth	11 (37.9)	22 (48.9)
Multiple pregnancy	1 (9.1)	1 (4.6)
Implantation rate	14/30 (48.3)	28/45 (62.2)
Babies born	12	23
Week of gestation (singletons)	38.2 ± 2.9	38.3 ± 2.8
Birth weight (cm)	3261 ± 576	3243 ± 706
Height (cm)	49.6 ± 1.8	49.4 ± 13.5
Sex ratio (male/female)	1	0.92
Minor malformation	1 (8.3)	1 (4.4)

Values in parentheses are percentages. Neonatal data are mean ± SD. All multiples were monochorial-diamniotic pregnancies. *P* > 0.05

## Discussion

Although AOA is a technique necessary for some patients to succeed in fertilization, embryo transfer, and pregnancy [33, 34], it is considered an add-on intervention [35]. However, since AOA techniques may overcome physiological problems in a rather “unphysiological” mode, caution should be taken and overuse should be avoided [18, 19].

In principle, AOA embryologists can choose from modified ICSI techniques [36], electrical [37, 38], or chemical activation approaches [39] to artificially activate oocytes or to stimulate mitosis [40, 41].

Many efforts have been made to emphasize that AOA, as it is applied today, can be considered a safe technique. In detail, gene expression of ICSI plus AOA was found to be closer to that in conventional IVF than with ICSI alone [42]. More reassuring evidence comes from an Italian group [43] that did not find an increased rate of chromosome segregation errors in meiosis II after exposure to 100 μM A23187 (which approximately corresponds to the tenfold concentration that is used in routine AOA). Others [44] reported that AOA did not increase the rate of aneuploid blastocysts compared to conventional ICSI. These results are in line with the present finding that irregular cleavages, potentially associated with aneuploidy [45], were not increased in ionophore-treated oocytes compared to their untreated counterparts.

More importantly, neurological development (cognition, language, motor skills) was not notably impaired in children aged 3–10 years [46]. Similarly, no increased rates of congenital birth defects were reported using AOA with calcimycin [47] or ionomycin [48]. The same holds true for the ready-to-use ionophore used in the present study since more than 130 healthy live births have been published [26, 27, 40, 41, 49, 50]. Although there is growing evidence supporting the clinical relevance of ionophores and their application in IVF laboratories, it must be clarified that AOA should not be considered a routine technique. Rather, it is an (still experimental) add-on technique that should only be used in case of a proper indication.

Annotation of morphokinetic parameters could increase the knowledge on safety aspects of this procedure. However, all AOA techniques that use data on morphokinetics are only available for chemical activation using ionophores. Preliminary published data are in disagreement regarding the potential effect of iono- and calcimycin on annotated morphokinetic parameters. Montag et al. [23] found that artificially activated oocytes (with ready-to-use calcimycin) and the associated embryos did not differ in terms of morphokinetics (t2PB to tB), while others [21, 22] did see such an association. In particular, t2PN [22], as well as tPNf, t3, and t5 [21], was found to be accelerated once ionomycin was applied for AOA.

Notably, the three preliminary studies mentioned above [21–23] analyzed only a limited number of AOA patients each (63 cycles in total). This flaw makes the only available original study on AOA and time-lapse imaging of particular interest. These authors [24] highlighted that the effect of ionomycin on the morphokinetics of (donated) oocytes and the associated embryos was minimal and limited to very early events such as t2PB and t3. They speculated that ionomycin produces a quicker increase in intracellular Ca^2+^ compared to sole injection of sperm alone. This temporal advance would then lead to an earlier inactivation of the maturation promoting factor, an earlier zink spark, earlier meiotic resumption, and, consequently, an earlier extrusion of the second polar body (t2PB). This hypothesis of our Spanish colleagues would further confirm that the first Ca^2+^peak is responsible for the oocyte response and drives further downstream effects, for example, CaMKII modulation [51].

To the best of our knowledge, this is the first study to analyze the potential association between ionophore usage and morphokinetics. In line with Martínez et al. [24], an accelerated series of events at very early stages of oocyte activation/fertilization was observed. In particular, in the present study, the shortening of the latency of the Ca^2+^ signal was reflected by an earlier t2PNa in the ionophore group. In contrast to the Spanish study [24], no further morphokinetic parameters up to tB were affected. More interestingly, this study suggests a more synchronized cleavage pattern in embryonic cell cycle 3 (referred to as s3), in which the individual blastomeres of the 4-cell embryo cleaved in an orchestrated manner until the 8-cell stage was reached. A shorter s3 is considered a positive biomarker and was found to be predictive of blastocyst formation, blastocyst quality, and implantation [52–55].

These contradictory findings may have been caused by the different ionophore types used. Nikiforaki et al. [56] observed a higher amplitude of the initial Ca^2+^ peak with ionomycin than with ready-to-use A23187, although the latency of the peak did not show obvious differences.

Alternatively, different study designs may have played a role. All the time-lapse literature cited above [21–24] used controls that were not cultured under identical (although comparable) culture conditions. We believe that a sibling oocyte approach could circumvent these limitations.

There were also differences in patient cohorts. Martinez et al. [24] exclusively focused on a patient with a poor prognosis and a known history of complete fertilization failure due to a mutation in the male PLCζ gene, while our patient group was composed of couples with suspected fertilization problems and, as such, represents a cohort known to have a better prognosis. No patients with complete fertilization failure were included in the present work because, in such a case, splitting of oocytes is obsolete and all gametes would have been treated with GM508 CultActive to minimize the risk of transfer cancelation. In other words, there is a chance that the initial Ca^2+^peak arises faster in patients with a substantial male factor, such as PLCζ gene mutations, while other patients with less severe impairments have a slightly longer latency of the calcium signal. The idea of “individualized” artificial oocyte activation, meaning that different medical indications might require different AOA approaches, is not new [49].

However, in light of the minimal changes in morphokinetics due to ionophores [24] and bearing in mind that a faster development at earlier stages (even if it would be conserved up to the blastocyst stage) is a rather positive sign [30], the present annotated dataset does support a relative innocuousness of ionophore treatment and, specifically, the ready-to-use ionophore GM508 CultActive [57, 58]. These results can assist in further evaluating the safety issues of AOA.
